# Biological activities, therapeutic potential, and pharmacological aspects of blackcurrants (*Ribes nigrum* L): A comprehensive review

**DOI:** 10.1002/fsn3.3592

**Published:** 2023-08-15

**Authors:** Afaf Ejaz, Sadaf Waliat, Muhammad Afzaal, Farhan Saeed, Aftab Ahmad, Ahmad Din, Huda Ateeq, Asma Asghar, Yasir Abbas Shah, Ahmad Rafi, Mahbubur Rahman Khan

**Affiliations:** ^1^ Food Safety and Biotechnology Lab, Department of Food Science Government College University Faisalabad Faisalabad Pakistan; ^2^ Department of Food and Nutrition Government College University Faisalabad Faisalabad Pakistan; ^3^ National Institute of Food Science & Technology University of Agriculture Faisalabad Faisalabad Pakistan; ^4^ Department of Food Processing and Preservation Hajee Mohammad Danesh Science & Technology University Dinajpur Bangladesh

**Keywords:** bioactive compounds, extraction techniques, functional properties, pharmacological, therapeutic

## Abstract

Blackcurrant possesses various health‐endorsing attributes owing to its polyphenol profile. Recent studies have demonstrated its therapeutic potential against various health disorders. Various bioactives present in blackcurrants have different functional and pharmacological aspects including anti‐inflammatory, antioxidant, and antimicrobial properties. The most dominant and important bioactive include anthocyanins, flavonols, phenolic acids, and polyunsaturated fatty acids. Food formats derived from blackcurrants comprise pomace, juice, powder, and extracts. All these food formats have industrial, prebiotic, and pharmacological benefits. In the current article, the nutritional composition, industrial applications, and therapeutic potential are discussed in the recent literature. Moreover, novel extraction techniques for the extraction of bioactive compounds present in blackcurrants and their safety concerns have been elaborated.

## Graphical abstract

Blackcurrant possesses various health‐endorsing attributes owing to its polyphenol profile. Food formats derived from blackcurrants have industrial, prebiotic, and pharmacological benefits.

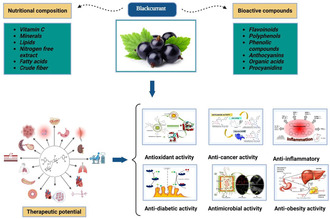

## 1 INTRODUCTION

*Ribes nigrum L*., generally called blackcurrant, is a flowering plant that contains deep purple, bittersweet, and seed‐bearing berries that can attain a diameter of about 1 cm (Gopalan et al., 2012). The berries (skin, flesh, and seeds), leaves as well as other plant parts can all be useful. The fruits can sometimes be eaten directly or produced in the form of jams, juices, and jellies. It is commonly used to synthesize dark violet pigments. Because of its enhanced antioxidant capacity and significant anthocyanin properties relative to a variety of fruits, blackcurrant (*Ribes nigrum* L.), a deep‐pigmented berry that is native to northern and central Europe and northern Asia, has become planted in many US regions. The separation, isolation, and classification of anthocyanins and other phenolic chemicals have been the primary objective of studies so far (Cacace & Mazza, 2003; Slimestad & Solheim, 2002).

Blackcurrants have been exposed to several anthocyanin extraction technologies. Furthermore, it is complicated to evaluate extraction efficiency over numerous technologies because a wide range of factors, particularly varieties and storage period, influence anthocyanin productivity (Nour et al., 2011; Rubinskiene, Jasutiene, et al., 2005; Rubinskiene, Viskelis, et al., 2005).

To improve the extraction efficiency of bioactive components, advanced innovations have been explored. These novel techniques comprise pulsed‐electric field extraction, microwave‐assisted extraction, ultrasonic‐assisted extraction, sonicated‐assisted extraction, and enzyme‐assisted extraction (Kaufmann & Christen, 2002). The removal efficiency of bioactive substances achieved using these approaches is equivalent to or even better than those achieved with traditional techniques, whereas they also require less solvent and need less time to finish.

Multiple biochemical compounds, like phenolic acids, organic acids, polyunsaturated fatty acids, and polyphenols, are abundant in blackcurrants (*Ribes nigrum* L.) (Delazar et al., 2010). Flavonols and anthocyanins are a couple of the polyphenolic components called blackcurrant flavonoids. Earlier findings have demonstrated that blackcurrants are a rich source of bioactive compounds (500–1342 mg/100 g of total polyphenols), predominately anthocyanins (160–411 mg/100 g) (Moyer et al., 2002). Natural plant dyes termed anthocyanins are found in a wide range of fruits, vegetables, and flowers. According to studies, blackcurrants comprise four anthocyanins: delphinidin‐3‐O‐glucoside, delphinidin‐3‐O‐rutinoside, cyanidin‐3‐O‐glucoside, and cyanidin‐3‐O‐rutinoside. Delphinidin‐3‐O‐rutinoside and cyanidin‐3‐O‐rutinoside are particular blackcurrant anthocyanins (Gopalan et al., 2012).

Numerous bioactive and flavoring components are vitamins, minerals, polyphenols, polyunsaturated fatty acids (PUFA), organic acids, vitamins (C,E), soluble and insoluble dietary fibers, tannins, and soluble sugars (Kowalski & de Mejia, 2021).

The antioxidant and chemical composition of blackcurrant were analyzed. Moisture, nitrogen‐free extract, crude fiber, crude protein, crude ash, and crude lipid correspondingly were the basic concentrations. P, K, and Ca were the mineral constituents. Moreover, blackcurrant has DPPH and ABTS radical scavenging characteristics. The blackcurrants FRAP and decreased powers were dependent on the dosage. Therefore, blackcurrant is a valuable source of essential nutrient elements, including organic antioxidants (Jeong et al., 2012). The quality, wholesomeness, and acceptance of dietary items produced from berries are eventually affected by phenolic compounds besides enhancing nutritional properties; it also improves the appearance and flavor of berries. These tiny round berries with incredibly flavorful seeds are generally used in processed items such as juice, jams and preservers, pie fillings, dessert toppings, yogurt, ice cream, mineral waters, teas, confectionery, and perfumes. It is also feasible to make gamma‐linolenic acid from seeds (Agrawal, 2011). The anthocyanin composition of the berries gives blackcurrant its rich purplish color. The presence of carbohydrates and acids controls the sweetness and sourness which alter sensory characteristics and customer satisfaction. Phenolic components increase tartness and saltiness, which can sometimes adversely affect the sensory quality and reduce customer perception of certain commodities (Laaksonen et al., 2012; Sandell et al., 2009).

Blackcurrants are now considered “super fruits” since they are supposed to have numerous therapeutic benefits, such as the capacity to treat chronic illnesses associated with oxidative stress (Lister et al., 2002). Several health‐promoting characteristics of blackcurrant compounds have been recognized, comprising immunomodulatory, antimicrobial, and anti‐inflammatory properties, reduction of reduced‐intensity lipoprotein, and a decline in cardiovascular disorders (Hurst et al., 2010). Moreover, blackcurrant juice and extract have been demonstrated to significantly slow the growth of Ehrlich carcinoma in vivo (Takata et al., 2005, 2007) and also to dramatically reduce the proliferation of prostate, stomach, intestine, and colon cancer cells, and breast in vitro (Boivin et al., 2007; Olsson et al., 2004). Anthocyanin‐rich blackcurrant extract was discovered to possess antioxidant, anti‐inflammatory, and immunostimulatory impacts in current clinical research.

## 2 NUTRITIONAL COMPOSITION

The blackcurrant berries are an excellent source of various phytochemical and aromatic constituents, such as minerals, polyphenols, vitamins, polyunsaturated fatty acids (PUFA), soluble and insoluble dietary fiber, tannins, soluble sugars, also organic acids and vitamins C and E (Kowalski & de Mejia, 2021). Nour et al. (2011)) research that citric acid is also abundant in blackcurrant fruit. Thus, according to Šavikin et al. (2013), linoleic acid was a highly abundant vital fatty acid, along with oleic and γ‐linolenic α‐linolenic stearidonic. Moreover, the n‐3/n‐6 important fatty acids percentage among overall researched blackcurrant varieties was varied from 0.27 to 0.36. Polyunsaturated fatty acids are dominantly found in blackcurrant seeds.

Conversely, decreased values of vitamin C were discovered by Rachtan‐Janicka et al. (2021)) (varies out of blackcurrant berries are renowned for their distinctly little sourness and bitter taste) (Archaina et al., 2018). Moreover, anthocyanins (ANTs) are abundantly present in them. Furthermore, significant health‐regulating characteristics of blackcurrant obtain from the occurrence of polyphenolic components which are secondary plant metabolites recognized by powerful antioxidant properties. Flavonoids and anthocyanins are essential polyphenols found in blackcurrant fruits.

The antioxidant properties and chemical composition of blackcurrant were analyzed. Moisture comprised 77.64%, nitrogen‐free extract comprised 17.41%, crude fiber comprised 3.08%, cured protein comprised 1.28%, crude ash comprised 0.31%, and crude lipid comprised 0.28%. The following were the basic concentrations. P (54.74 mg/100 g), Ca (26.45 mg/100 g), and K (177.36 mg/100 g) were the mineral constituents shown in Table 1. Moreover, blackcurrant has DPPH and ABTS radical scavenging characteristics. The blackcurrants FRAP and decreased power depended on dosage. Therefore, blackcurrant is a valuable source of essential nutrient elements, including organic antioxidants (Jeong et al., 2012).

**TABLE 1:** Nutritional composition of blackcurrant.

Components	Yield	Blackcurrant fraction	References
Nitrogen‐free extract	17.41%	Fresh fruit	(Jeong et al., 2012)
Lipids	4.1%	Pomace	(Sójka & Król, 2009)
Moisture	7.5%	Pomace	(Alba et al., 2018)
Ash	0.31%	Fruit	(Mattila et al., 2011)
Fiber	3.08%	Fruit	(Cho et al., 2010)
Protein	17%	Press cake	(Hilz et al., 2005)
Total organic acids	95%	Leaf	(Mikulic‐Petkovsek et al., 2013)
Essential fatty acids	0.27%–0.36%	Seeds	(Nour et al., 2011)
Vitamin C	60%	Jam	(Viberg et al., 1997)
Minerals			
Ca	26.45 mg/100 g	Fruit	(Kim & Shin, 2011)
P	54.74 mg/100 g	Fresh fruit	(Jeong et al., 2012)
Mg	4233 μg/g dw	Leaves	(Niskanen, 2002)
K	8700 μg/g dw	Leaves	(Nour et al., 2014)
Trace minerals	19.5–82 μg/g dw	Leaves	(Niskanen, 2002)

They are manufactured in a great variety of items (such as jams, jellies, dry fruits, alcoholic and non‐alcoholic beverages, juices, and fruit nectars) because these are a high source of pectin, carbohydrates, and aromatic components (Cortez & Gonzalez de Mejia, 2019; Sokół‐Łętowska et al., 2014). Furthermore, the fruit remains of blackcurrant (such as from juice production) are utilized to create organic colorants or as a source of biologically active compounds for the manufacturing of various healthy food items (Gagneten et al., 2021). The minerals, significantly magnesium, potassium, calcium, and iron are abundantly found in blackcurrant fruits.

Calcium was the mineral discovered in the greatest concentration, preceded by magnesium and potassium, and sodium was contained in much lesser quantities. The levels of potassium were significantly increased relative to sodium; therefore, the K/Na percentage was incredibly increased. Considering foods with a significant K/Na ratio was associated with a decreased risk of hypertension, this is advantageous from the perspective of nutrients. Moreover, Nour et al. (2014) also discovered that the significant concentrations of magnesium and calcium had been found in blackcurrants.

## 3 EXTRACTION OF BLACKCURRANT BY DIFFERENT NOVEL TECHNIQUES

**FIGURE 1:**
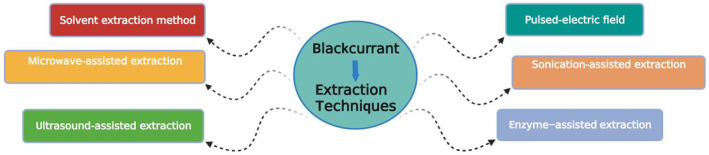
Different extraction techniques used for blackcurrant.

Extraction is a typical method for extracting bioactive components from plant parts, including the tissues of blackcurrants. The necessity for expensive, prolonged extraction intervals, better quality solvent, the evaporation of substantial concentrations of solvent, low extraction discrimination, and the heat disintegration of heat‐labile compounds are the primary problems of traditional extraction methods (Selvamuthukumaran & Shi, 2017). To get over these constraints, innovative and effective extraction technologies were developed. Advanced techniques have been investigated alongside traditional solvent extraction technologies to increase the output of bioactive components. These novel techniques such as ultrasonic‐assisted extraction, sonicated‐assisted extraction, enzyme‐assisted extraction, pulsed electrical field extraction, and microwave‐assisted extraction (Kaufmann & Christen, 2002) (Figure 1). These methods need less solvent intake and decrease extraction duration, overall the extraction values of bioactive phytonutrients are equivalent to or possibly increased than those produced by applying traditional techniques.

### 3.1 Solvent extraction method

Phytochemical compounds are generally obtained by ethanol, acetone, water, and/or methanol. The most accurate method to extract anthocyanins from blackcurrants is methanol and ethanol rather than water (Lapornik et al., 2005). Before treatment, the blackcurrant berries remained preserved. The standard process was employed to synthesize ethanol extracts: 600 g of fruits were weighed out and kept in a glass jar, soaked in 1 L of hard liquor (40%, 60%, or 96% of food‐graded ethanol), letting sit for 3 weeks. The samples were purified after this duration and instantly examined, a blend of various solvents exhibited improved extraction capability than a simple solvent (Nour et al., 2013).

The utilization of methanol and acetone in food is prohibited even though they function as the best extraction method (Harbourne et al., 2013). Seed remnants were separated three times at room temperature with 50% acetone (3 [10] mL solvent/g of flour) (Bakowska‐Barczak et al., 2009). Phytochemicals were obtained by blending 10 g of frozen fruits for 30 s in 70 mL of 80% aqueous methanol including 0.1% formic acid (Bakowska‐Barczak & Kolodziejczyk, 2011). Moreover, the availability of acid regulates anthocyanin extraction yield by enabling those bioactive components from blackcurrant skin and pomace (Azman et al., 2020).

The cake was kept in the freezer before extraction although the fruit juices were obtained by using a basic juicer. Hot water oxidized with 0.2% citric acid was applied to extract the colored compounds from the blackcurrant cakes for 3 h at 60°C (Rubinskiene, Jasutiene, et al., 2005; Rubinskiene, Viskelis, et al., 2005). It is important to keep in mind that acylated anthocyanin may be decomposed by hydrochloric acid.

### 3.2 Microwave‐assisted extraction

Among the most frequently used extraction techniques nowadays in treating pesticides, organic pollutants, phenols, polymers, medicines, and natural products is microwave‐assisted extraction (MAE). It could be a viable option for traditional methods, because of the faster extraction process and reduced solvent consumption. To enable faster extraction of essential and hydrophilic chemicals from the sample into the extraction solvent, microwave energy is consumed in this type of extraction to melt solvents in association with samples (Morales‐Muñoz et al., 2006). According to the polar nature of water molecules, microwave radiation can be collected properly. Hence, in the MAE technique, water is an accurate and environmentally sustainable solvent. Although water interacts more effectively with microwaves than traditional organic solvents because of its large dielectric constant and efficient loss factor, in some conditions, organic substances' permeability is reduced.

The extraction process was take place at a regular temperature of 80°C. Aqueous HCL at pH 2, citric acid solution at pH 2, and solution including 50 ppm SO_2_ and 1% citric acid were solvents employed. 100 g of blackcurrant crush leftover was utilized as a sample batch. During the process, the microwave energy varied from 140 to 700 W, the solvent pH varied from pHs2 to 7, the solvent ratio of marc varied from 1:10 to 1:20, and the separation duration varied from 10 to 30 min (Pap et al., 2012).

### 3.3 Ultrasound‐assisted extraction

While conducting ultrasonic‐assisted extraction (UAE), a strong‐intensity zone of ultrasound (10–1000 W/cm^2^) is utilized. Mass exchange and solvent solubility were easily achieved by UAE. As a consequence, it decreases the quantity of solvent intake and extraction temperature while enhancing the extraction yield and speed (Virot et al., 2010). In contrast with traditional extraction techniques, UAE improves the volume and persistence of polymeric anthocyanins in fruit extracts abundant in anthocyanin (Wang et al., 2016).

Ultrasound processing together with traditional extraction provided significant findings according to the types of blackcurrant samples used: frozen, lyophilized, and over‐dried (Oancea et al., 2014). Through an ultrasonic water bath, samples of leaves were recovered with the application of ultrasound. The plant sample (10 g) was kept in a volumetric flask and 100 mL of distilled water was used. For one hour, the mixture was incubated. A rotary evaporator was applied to eliminate the extract solvents, and the solution was then dried at 60°C. The produced dry extract was kept in the glass bottles at 4°C before the examination.

At 25°C, the blackcurrant pomace was blended 1:10 (w/v) with distilled water. The blend was kept in the ultrasound bath. For 2 h, the ultrasound of 27 kHz frequency and 6 W/cm^2^ density was employed. Air bubbles were incorporated into the solution during the sonication technique to allow faster extract decomposition (Vorobyova et al., 2020).

By utilizing 96% ethanol as a solvent, the fruit samples (10.0 g) were obtained. The extraction process took place using an ultrasonic bath, which operated at room temperature for one hour. Following purification, 5 mL of the liquid powder was utilized to evaluate extraction concentrations. A rotary evaporator was applied to evaporate the solvent through a vacuum and dried at 30°C to stable weight (Paunović et al., 2017).

### 3.4 Pulsed‐electric field

Pulsed‐electric field processing is a non‐thermal technique that involves establishing an electric field and treating the sample to multiple low‐voltage pulses of moderately intense although significantly lower energy (Bobinaitė et al., 2015). Whenever pulsed‐electric field is administered to plant tissues, the outcome is a cell membrane permeabilization that also stimulates the discharge of fluid and essential elements from the cell (Barba, Parniakov, et al., 2015). Pulsed‐electric field‐assisted extraction accelerates mass transport despite treating the material to very high temperatures, minimizing the decomposition of thermally sensitive components (Barba, Galanakis, et al., 2015). Furthermore, this method takes to reduce energy utilization and fast processing duration in contrast with traditional extraction techniques rendering it a particularly compelling technique for the food company (Frey et al., 2017; Vorobiev & Lebovka, 2017). As far as we are familiar, still, one research examined how pulsed‐electric field treatment influences blackcurrant anthocyanins extraction. It indicated that the quantity of all monomeric anthocyanins obtained from blackcurrants improved by approximately 6% given suitable treatment parameters (Gagneten et al., 2019). Regarding the insufficient literature on pulsed‐electric field treatment, additional research is necessary to understand how pulsed‐electric field improves blackcurrant anthocyanins extraction.

60 g of fruit were put in custom‐designed, lab‐scale container that was a 21 cm × 5 cm × 1 cm acrylic cuvette. The treating container was placed in ice to reduce the temperature impact for the relevant investigations. A square wave electroporator was utilized to perform the electroporation treatment. Using a strong‐voltage sensor (100×) and a shunt resistor (1 ohm) in combination with the container, as well as two channels, along with an oscilloscope 100 MHz, the pulse current and voltage were evaluated. These evidences were documented throughout the duration of procedure. For all the investigations, the pulse width was 100 milliseconds (Gagneten et al., 2019).

### 3.5 Sonication‐assisted extraction

The research proposes sonication‐assisted extraction (SAE) as a rapid and efficient method to extract essential components, such as polyphenols, from complex living organisms containing a wide range of chemical compounds, including primary and secondary metabolites (Belwal et al., 2020; Şahin et al., 2015). Ultrasonic is a form of sound wave that impacts gases, solids, and liquids physically at a wavelength greater than 20 kHz (Tiwari, 2015). Initially, vibration frequency converts into physiological pressure, which further transmits power to the medium. The energy is then transmitted from the medium to the substance in interaction with the wave (Fu et al., 2020). Because of its low cost, ease of use, and high efficiency, this extraction system (SAE) is an ideal substitute for conventional approaches (Al‐Dhabi et al., 2017).

The basic body undergoes sonication and acoustic cavitation to provide UAE impacts (Tiwari, 2015). By developing alternating pressure regions in the material, sonic waves generate gas bubbles. These gas bubbles immediately condense at critical points liberating the energy they possess. This produces shock waves with considerable energy and heat, which eventually influence the sample physically (Chemat et al., 2017; Pico, 2013).

Thus, according to Nour et al. (2014), all of the preceding extraction methods were employed to triplicate leaf extracts samples (2.5 g) out of each cultivar: (a) an extraction in 100 mL of distilled water at 80°C for 15 min; (b) an extraction with sonicated‐assisted in 100 mL 40% ethanol at 20°C for 55 min; (c) an extraction with sonicated‐assisted in 100 mL 80% ethanol at 20°C for 55 min. The supernatants from the extraction were separated through a 0.45 m filter and preserved at 4°C till evaluation after being centrifuged at 3000g/for 10 min.

### 3.6 Enzyme‐assisted extraction

Enzyme‐assisted extraction (EAE) depends on the capability of specific enzymes to decompose or destroy the cell membranes to liberate substances (Akyüz & Ersus, 2021; Meini et al., 2019). Various enzymes, including protease, xylanase, pectinase, cellulase, α–amylase, and β–glucosidase, have previously been used to recover bioactive components from plants (Shen et al., 2021). Pectinases are the most frequently utilized enzymes across all of them for removing natural components from plant matrices because of their broad substrate selectivity and strong stability during unfavorable situations (Abdullah et al., 2021). Blackcurrant plant cell membranes are substantially decomposed by a spectrum of pectinolytic enzyme preparations and protease during enzyme hydrolysis. During the maceration procedure, blackcurrant pulp specimens were exposed to several pectinolytic enzyme preparations; a few of them dramatically increased the anthocyanin concentrations in blackcurrant juice, whereas others had negligible influence (Landbo & Meyer, 2004).

EAE possesses significant advantages over traditional techniques for obtaining anthocyanins because it is a relatively easy process that usually does not need chemical solvents, and because it consumes minimal energy, time, or temperature to obtain useful extraction content (Domínguez‐Rodríguez et al., 2021). EAE was utilized to obtain anthocyanins and phenolic compounds from several matrices, particularly Akebia trifoliate flowers, monguba, dragon fruit, and wild strawberries (Zhang et al., 2019).

Uniform 0.2 g samples were transferred to 15 mL solvent, shaken at 100 rpm for 20 min, and then recovered. A pectinase from Aspergillus niger was the enzyme tested for recent research. The parameters are chosen, and the values for each were temperature (30, 45, and 60°C), pH of the solvent (4, 5, and 6), % ethanol in solvent (10, 35, and 60%), and enzyme unit per g of sample (100, 550, and 1000 U/g) (González et al., 2022).

The blackcurrant fruit diluted solution (20.0 g) with 2% enzyme mix (papain: pectinase = 2) was placed in a 500 mL beaker, and then a buffer solution (pH = 5.3) was introduced at 20:1 mL/g. The polysaccharides were subsequently isolated at 40°C using an ultrasonic cell disintegrator for 45 min, and the ultrasonic power was 600 W (Xu et al., 2016).

## 4 EXTRACTED BIOACTIVE COMPOUNDS

**FIGURE 2:**
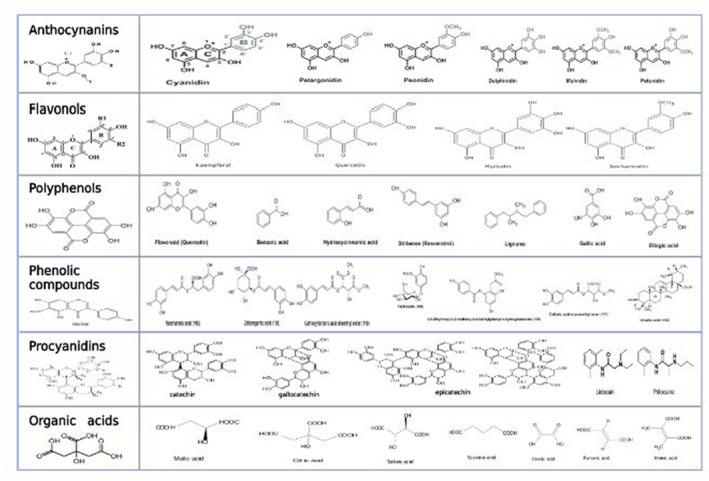
Chemical structures of bioactive compounds extracted from blackcurrant.

Blackcurrant contains a significant amount of phenolic compounds named anthocyanin, which makes up nearly 80% of all phenolic compounds. Approximately, 97% of the anthocyanin in blackcurrants derived from the four basic components delphinidin‐3‐O‐glucoside, delphinidin‐3‐O‐rutinoside, cyanidin‐3‐O‐glucoside, and cyanidin‐3‐O‐rutinoside (Anttonen & Karjalainen, 2006). Slimestad and Solheim (2002) discovered 15 anthocyanins structures found in the extracts of blackcurrant berries as a result of their current research into anthocyanin components of blackcurrants (Figure 2). Numerous researches have examined the several polyphenolic components of the fruits such as the analysis of anthocyanins in rose wine made from cv. Öküzgözü Grapes. Thirteen different anthocyanins, including five acetyl glucosides, five glucosides, and three coumaroyl glucosides, were found and quantified. The presence of a significant amount of malvidin‐3‐glucoside and its acylated esters was discovered to be a key element of Öküzgözü rose wine (Kelebek et al., 2007), and, to a limited extent, other plant parts, including leaves and buds, have recognized the increased phenolic component levels in blackcurrants (Oszmianski et al., 2011; Raudsepp et al., 2010). The distribution of the anthocyanin and other phenolics was determined in the dried skin (Azman et al., 2021), dried press cake (Grimm et al., 2020), from the clarified and concentrated juice (Pap et al., 2010, 2012), and the extracts of blackcurrant fruits and powders (Hui et al., 2020; Zhao et al., 2021). The amounts of anthocyanins *Ribes nigrum L* plant develop across the whole ripening stage; the over‐ripe berries had increased levels of pigments. Ben Alder and Vakariai acquired the maximum anthocyanins levels of five examined blackcurrant cultivars. The breed and ripening duration influence the percentage of the reported anthocyanins. It varied among cy‐3‐rut (36.6%–53.0%), del‐3‐rut (28.3%–39.1%), cy‐3‐glc (4.5–11.9%), and del‐3‐glc (8.0%–20.3%) (Rubinskiene, Jasutiene, et al., 2005; Rubinskiene, Viskelis, et al., 2005).

Ascorbic acid, among the non‐phenolic compounds in blackcurrant, may improve the liquor's capacity to eliminate free radicals. In the current research, Borges et al. (2010)) discovered 11 delphinidin‐, cyanidin‐, malvidin‐, petunidin‐, and protein‐based anthocyanins, the major elements including delphinidin‐3‐O‐glucoside, delphinidin‐3‐O‐rutinoside, and cyanidin‐3‐O‐rutinoside. They determined that while vitamin C accounted for 18% of the whole antioxidant properties, blackcurrants anthocyanins represented 73% of it. Anthocyanins quantities in blackcurrant ethanolic extracts ranged from 198 to 922 mg/L, whereas the total phenolics values ranged from 1261 to 1694 mg GAE/l (Nour et al., 2013) (see Table 2).

**TABLE 2:** Extracted bioactive compounds of blackcurrant.

Bioactive compounds	Cultivar	Blackcurrant fraction	Extraction method	Solvent used	Yield	References
Polyphenols	Ben Nevis	Fruit	Solvent extraction	Acetone	815 mg/100 g	(Moyer et al., 2002)
Procyanidins	Ben Alder	Pomace	Sonicated‐assisted extraction	Methanol	215 mg/100 g	(Bakowska‐Barczak & Kolodziejczyk, 2011)
Flavonol Glycosides	Mortti	Fruit	Solvent extraction	Ethyl acetate and methanol	10.24 mg/100 g	(Zheng et al., 2012)
Anthocyanin	Ben Nevis	Juice	MAE	HCl and citric acid	16.9 mg/g	(Pap et al., 2013)
Antioxidant activity	Titania	Juice	Pulsed‐electric field	Water	1.67 mg/g	(Gagneten et al., 2019)
Total phenolic content	Blackdown	Leaves	Sonicated‐assisted extraction	Ethanol	35.34 mg/g	(Nour et al., 2014)
Carbohydrates	Ben Lomond	Pomace with seeds	Enzyme‐assisted extraction	Water	0.818 mg/mL	(Landbo & Meyer, 2001)
Total monomeric anthocyanins	Ben Tron and Ben Nare	Clarified juice	Ultrasonic‐assisted	Acetone	144 mg/100 g	(Holtung et al., 2011)
Flavonoid	Ben Sarek	Frozen fruit	Solvent extraction	Methanol	11.50 mg/100 g	(Bakowska‐Barczak & Kolodziejczyk, 2011)
Flavonols and flavan‐3‐ols	Tsema	Fruit	Ultrasonic‐assisted	Ethanol	7.43 mg/100 g	(Paunović et al., 2017)

The phenolic concentrations in the blackcurrants demonstrated maximum values of total polyphenols in fruits, 955 mg/100 g FW in total (Bordonaba & Terry, 2008). Moyer et al. (2002) identified equivalent levels of total polyphenols for “Ben Nevis” variety (815 mg/100 g FW) and decreased levels of total polyphenols for “Ben Conan” variety (498 mg/100 g FW).

The thiolysis technique was used to determine the procyanidins levels in blackcurrants. Epicatechin benzylthioether and (epi) gallocatechin benzylthioether were liberated as the free flavan‐3‐ols and free flavan‐3‐ols adducts, correspondingly, by the thiolytic breakdown of blackcurrant procyanidins. The contents of procyanidins in blackcurrant berries range from 140 mg/100 g FW for “Ben Conan” to 267 mg/100 g FW for “Ben Sarek” (Wu et al., 2004).

The flavonols quercetin, myricetin, and kaempferol were identified in six major conjugates. Earlier studies have discovered numerous flavonol conjugates in the fruits of blackcurrants, containing rutinoside (rhamnosylglucose), galactose, malonylglucose, arabinose, and glucose (Sandell et al., 2009).

Blackcurrants have a powerful antioxidant activity that has been earlier reported (Ehala et al., 2005), and their significant antioxidant properties are linked with their high percentage of phenolic components. Depending on the scavenging action of the stabilized free radicals ABTS and DPPH, the antioxidant capacity was evaluated. The variety identified as “Ben Alder” had the strongest antioxidant action averaging 4.5 and 2.4 mM/100 g FW, for DPPH and ABTS correspondingly. “Ben Conan” demonstrated the reduced antioxidant properties among the varieties tested, 3.7 and 2.1 mM/100 g FW, for ABTS and DPPH correspondingly (Bakowska‐Bakowska‐Barczak & Kolodziejczyk, 2011).

Specific sugars, organic acids, and vitamin C were evaluated as primary metabolites in tested blackcurrant varieties. Fructose and glucose were the two main sugars, while sucrose was second. The overall quantity of the two dominant sugars comprised about 78 and 93% of the whole sugar content (Zheng et al., 2009). The other similarly seemed to have decreased the number of carbohydrates. Blackcurrant varieties predominantly including citric and malic acids, which both accounted for approximately 95% of total organic acids (Milivojević et al., 2010). The blackcurrant fruit is a good source of vitamin C (Szajdek & Borowska, 2008). Various blackcurrant varieties reported vitamin C levels that varied from 116 to 342 mg 100 g^−1^ fruit.

The number of glycosides of myricetin, kaempferol, and quercetin was also similar to earlier research (Milivojevic et al., 2012). The major constituent of a flavonoid molecule is generally the aglycone, and significant diversity in their glycosides has been found in blackcurrant species. The percentage of flavonols and Kaempferol glycosides varied from 38% to 75%; the findings demonstrate that cultivars had a very increasing proportion of kaempferol3‐galactoside and kaempferol‐3‐rutinoside in their fruit. Myricetin glycosides from among 7% to 28% of the total flavonol tested were myricetin glycosides, and 18% to 34% were quercetin glycosides (Mikulic‐Petkovsek et al., 2013).

The numerous plant segments include significant flavones like compounds of quercetin, myricetin, kaempferol, and isorhamnetin. Most of the time the flavones originate as catechin (flavon3‐ols) or as polymers. Blackcurrant comprises flavan‐3‐ ols catechin, epicatechin, epigallocatechin, and their galloyl variants more extensively (Sójka & Król, 2009). The unsaturated fatty acids, stearidonic acids, and γ‐linolenic, which perform an essential function in humans as precursors of certain long‐chain polyunsaturated fatty acids including hormones, are understood to be found in blackcurrant seed oil (Guil‐Guerrero, 2007).

## 5 FOOD FORMATS AND TECHNOLOGY

### 5.1 Blackcurrant pomace

Commercial seedless blackcurrant pomace is an outstanding source of dietary protein and essential fiber. Because of high levels of polyphenols available, seedless blackcurrant pomace shows considerable antioxidant effect. While using industrial pomace, a greater standard of component diversity should be recognized. The producing season and juice extraction technique influence the pomace composition (Sójka & Król, 2009). Throughout the extraction of blackcurrant extract, a significant level of pomace in blackcurrant formed. As an industrial effluent, discarding pomace is always an environmental challenge. Kunachowicz et al. (2005) observed that pomace exhibited higher levels of lipid and protein constituents compared to the dry matter of the fruits. The research demonstrated that decreased amounts of sugars available in the pomace identified the majority of sugars, approximately 96%, transfer to the juice throughout the production chain. In the same manner, as sugars are removed from the fruit and introduced to juice during processing, the alkalinity of blackcurrant extract, evaluated by dry mass, is around 92% (Rubinskiene, Jasutiene, et al., 2005; Rubinskiene, Viskelis, et al., 2005; Siksnianas et al., 2006). The major organic acid found in blackcurrant pomace was citric acid, although malic acid was also identified.

### 5.2 Blackcurrant extract

To effectively liberate bound phytochemicals from wastes, the extract is dependent on chemical and physical facilitation of extraction. Blackcurrant extract contains more comprehensive and dense phytochemical constituents than the conventionally manually extracted juice (Archaina et al., 2017). Multiple nutritional substances, particularly phenolics and anthocyanins, were obtained from blackcurrants through their maceration in alcohol. The amount of anthocyanins, overall phenolics, and antioxidant action of blackcurrant ethanol extracts were affected by ethanol level and blackcurrant genotype. Hence, the total anthocyanin concentration including antioxidant effect was maximum at 60% ethanol level, whereas overall phenolics quantity was maximum at 96% ethanol level (Nour et al., 2013). Furthermore, only with blackcurrant juice or juice concentrate, extraction chemicals are usually undesirable for preparing a wholesome diet. Moreover, blackcurrant extract is abrasive, sour, and volatile in free forms, which restricts how extensively it may be employed in the production of blackcurrant beneficial food commodities (Cortez & Gonzalez de Mejia, 2019).

### 5.3 Blackcurrant powder

Through freeze‐drying method, it is synthesized. The freeze‐dried blackcurrant extract is also crushed into granules, including the overall nutritional composition of the actual blackcurrant fruit. Because of the blackcurrant dietary fiber, the unprocessed blackcurrant is just slightly soluble in water. This could restrict it from being applied in aqueous solutions (Mofasser Hossain et al., 2017). Hence, the nutrient‐dense insoluble particles could be integrated with solid food substrates, especially dough, to add phytonutrients, color, and dietary fiber. Additionally, past research from which blackcurrant powder was first introduced to biscuit products to make beneficial snacks demonstrated that it could replace the flours.

### 5.4 Blackcurrant juice

For storage, natural blackcurrant berry is generally produced into juice or juice concentrate and is subsequently manufactured into dietary items that include blackcurrants (Woodward et al., 2011). Anthocyanin as well as polyphenols (the combination of anthocyanin, flavonols, proanthocyanidins, and phenolic acids) is particularly plentiful in freshly made blackcurrant juice (Landbo & Meyer, 2004). However, a dramatically reduced amount of anthocyanin has been discovered in industrial blackcurrant juice (Hollands et al., 2008). This can be described in two manners: initially, many industrial blackcurrant juices are solubilized; and second, the polyphenol concentration of blackcurrants can be lowered through numerous processing techniques used to generate commercial juices including heating, crushing, pressing, pasteurization, clarification, filtration, and enzyme treatment (Koponen et al., 2008). Moreover, throughout preservation, substantial loss of anthocyanins can be detected. The prepared blackcurrant juice concentrate is recognized for its abrasive and sour taste. Consequently, a considerable volume of sugar was introduced to the concentrate during processing to disguise these undesirable flavors (Cortez & Gonzalez de Mejia, 2019).

### 5.5 Industrial applications

**FIGURE 3:**
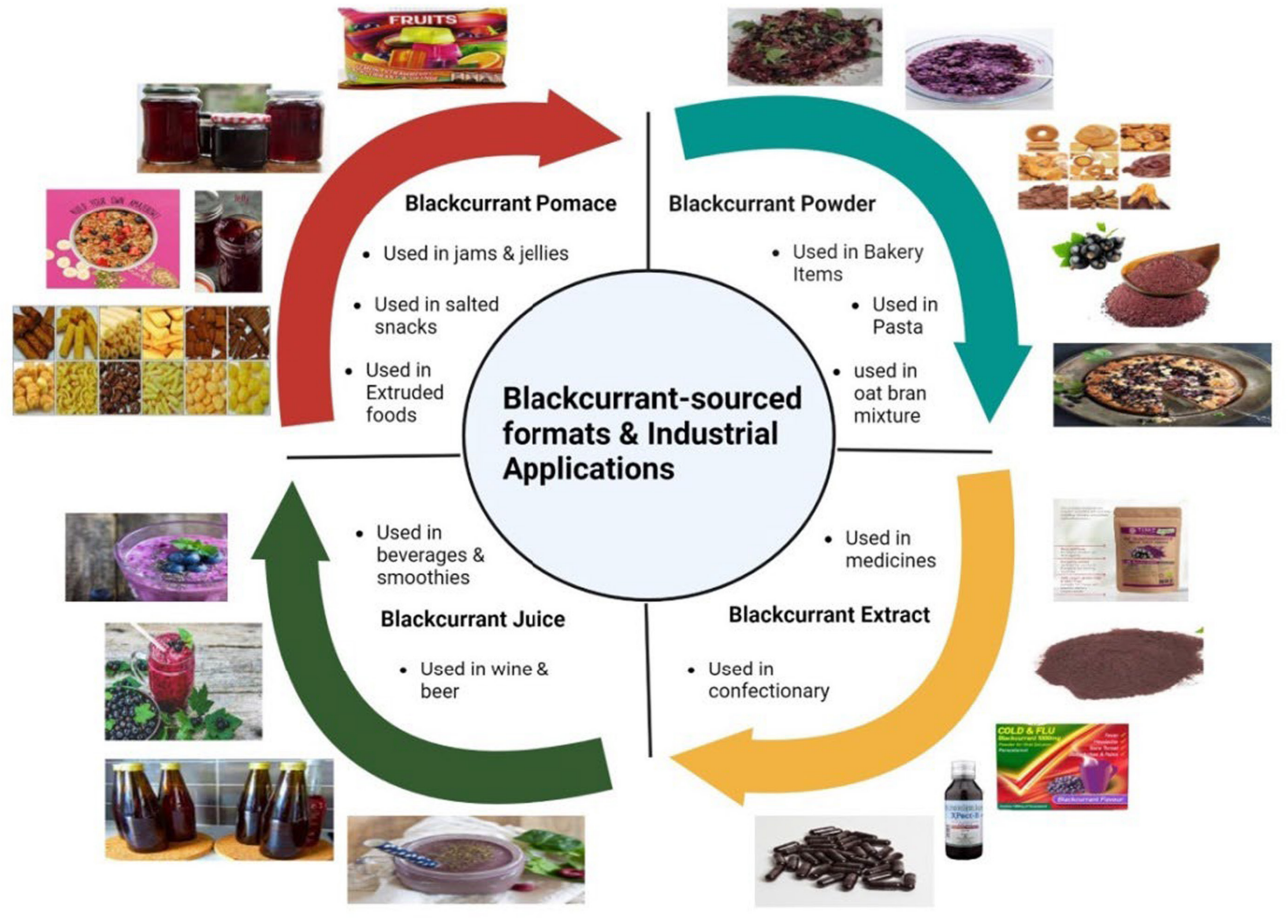
Blackcurrant‐sourced formats and industrial applications.

The eventual aim of numerous food layouts is to integrate them within marketable food items. It is viable to produce valuable food items utilizing blackcurrant pomace powder, blackcurrant powder, and blackcurrant encapsulate (Figure 3). Due to their antibacterial properties, blackcurrant flavonoids have been esteemed predominantly by the food and pharmaceutical industries as well as by the packaging industry (de Araújo et al., 2021; Yousefi et al., 2021). Every kind of material possesses its distinctive features. It is important to investigate their implementation independently. The latest investigation has placed a significant amount of attention on blackcurrant powder. Blackcurrant powder was incorporated into bakery items by Mofasser Hossain et al. (2017) and also used in pasta items (Bustos et al., 2020). Blackcurrant powder has been incorporated by Hui et al. (2021) into the oat bran mixture. Furthermore, powdered blackcurrant pomace that has been treated has been added to a wide range of food. Mäkilä et al. (2014) integrated blackcurrant pomace into extruded food products. Blackcurrant pomace will be utilized to prepare salted snacks (Schmidt et al., 2018). These integrations minimized the degradation of palatable raw material constituents and enhanced the nutritional properties of final products, due to their increased dietary fiber and bioactive compositions. Just a few research has examined the usage of blackcurrant supplements. Applying whey protein concentrate as such wall material, Wu, Hui, Mu, et al. (2021) and Wu, Hui, Stipkovits, et al. (2021) effectively built useful blackcurrants encapsulation in a diet‐compatible pathway. To produce the beneficial food items generated from encapsulates, the scientist then paired these therapeutic blackcurrant encapsulates with a natural food matrix (Wu, Hui, Mu, et al., 2021; Wu, Hui, Stipkovits, et al., 2021). Their findings presented an alternative for all possible developments of fruit‐sourced extracts or concentrates.

### 5.6 Encapsulation technology

Most of the polyphenols in blackcurrants are flavonols and anthocyanins. The polyphenols in blackcurrants remained in place throughout 9 months of freezing treatment. The extended storage life of blackcurrants is achieved by the bioactive substances' strong level of stability under freezer retention (Bakowska‐Barczak & Kolodziejczyk, 2011). Multiple studies on the persistence of polyphenols, mainly anthocyanins, have indicated that they are influenced by variables including metal ions, pH, light exposure to oxygen, temperature, and enzymatic actions (Bąkowska et al., 2003). The durability is a significant factor to keep in mind when utilizing polyphenols as food colorants and antioxidants. Encapsulation methods, like drying (freeze‐drying and spray‐drying) (Ersus & Yurdagel, 2007), emulsification, liposomal encapsulation, gelatin, and complexation, might boost the durability of polyphenols (Zuidam & Shimoni, 2010). The approach of microencapsulation includes packing the delicate materials inside of a coating or wall material. The barrier layer controls substance release while preserving the critical components from undesirable responses. Anthocyanin and phenolic components are enveloped in maltodextrin and inulin (Ersus & Yurdagel, 2007; Saénz et al., 2009). The most effective method to enclose the bioactive components in anthocyanin with macromolecules (carbohydrates, protein, etc.) is drying, including spray‐ and freeze‐drying (Mahdavi et al., 2014). Although freeze‐drying is the appropriate method to encapsulate temperature‐sensitive bioactive like anthocyanins, spray‐drying is commonly accessible and simple to industrialize. Under a vacuum drying temperature of 90°C, inulin was demonstrated to have preventive properties on blackcurrant anthocyanins (Michalska et al., 2019). For a wide range of food purposes, particularly cookies and pasta, the encapsulated blackcurrant extract might be utilized as organic colorants, reservoir of bioactive constituents, and proteins.

## 6 THERAPEUTIC POTENTIAL

**FIGURE 4:**
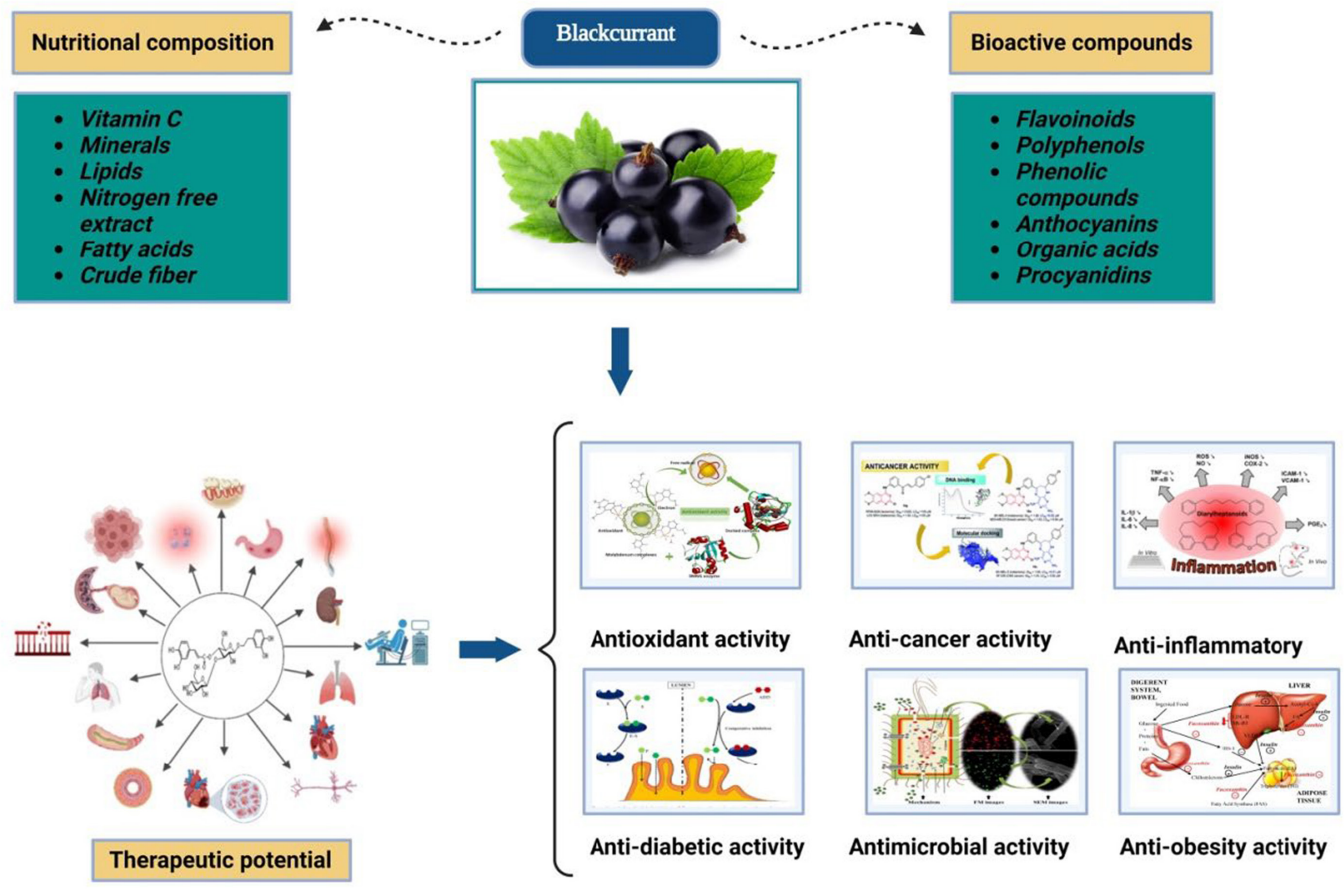
Therapeutic potential of blackcurrant.

Blackcurrant has a long tradition of a secure application, as well as being thoroughly investigated for its significant health effects. Limited research has focused on the additional therapeutic properties of blackcurrant, like vasculoprotective properties (Horie et al., 2021), hypocholesterolemic impact (Nanashima et al., 2021), improvement in the oxidation of fat (Song et al., 2021), regulation in brain functioning (Gibson et al., 2020), gut microbiota regulation (Song et al., 2021), and decrease in postprandial blood sugar (Iizuka et al., 2018), in addition to these broadly researched therapeutic properties (e.g., anti‐inflammatory, neuroprotective activities, anti‐cancer impact, and antioxidant) (Figure 4). The results indicate that polyphenols exhibit antioxidant activity that reduces the probability of developing various non‐communicable chronic conditions (chronic inflammatory disorders, atherosclerosis, type 2 diabetes, neurodegenerative disorder, and certain type of cancer) (Serino & Salazar, 2018). Taking fresh and processed blackcurrant (like juices and items comprising fruit extract) may assist with the nutritional treatment of the cardiovascular disorder, eye aliments, and obesity maintaining blood's normal lipid composition (Khoo et al., 2019; Lee et al., 2017, 2019). The ideal balance of unsaturated fatty acids abundant in fruit seed could also contribute to the management of autoimmune disorders (Cameron et al., 2009). The major recently found therapeutic properties of blackcurrant compounds have been summarized in Table 3.

**TABLE 3:** Therapeutic potential of blackcurrant.

Main health benefits	Blackcurrant formats	Pharmacological effect	References
Cardiovascular pathway	Juice	Stimulated paraoxanase 1 activity, regulating macrophage cholesterol retardation	(Rosenblat et al., 2010)
	Extract	Enhanced endothelial NO synthase function and blood vessels expansion	(Edirisinghe et al., 2011)
Vasculoprotective impact	Extract	Diabetes patient's blood vessels staying intact has a positive impact on their health	(Horie et al., 2021)
		High calcium flux restoration in type‐1 muscarinic R's	(Joseph et al., 2004)
Exercise and immunity restoration	Anthocyanin's extract	Significant impact on inherent immunity and post‐workout relaxation	(Hurst et al., 2019)
Ocular system	Juice extract	Increased dark adaptation and rhodopsin synthesis through improved rhodopsin precursor production	(Matsumoto et al., 2006)
	Extract	In artificially created myopia, these proportions of globe constituents parts were not permitted to increase	(Iida et al., 2010)
Changes in blood flow	Extract	Increasing forearm blood circulation during prolonged times of sitting	(Barnes et al., 2020)
		Lower elderly individual's systolic and diastolic ambient blood pressure	(Cook et al., 2020)
		Consumption of NZBC for more than a brief period of time decreases central blood pressure and central arterial stiffness in aged persons	(Okamoto et al., 2020)
Skeletal system	Leaf extract	Minimized osteoarthritis degradation	(Garbacki et al., 2002)
	Seed oil	Lowered intensity of pain intensity and enhanced disability	(Cameron et al., 2011)
Hypocholesteoremic impacts	Extract	Decrease the amount of hydroxymethylglutary‐CoA reducates, limits the intake of cholesterol, and stimulates the absorption of low‐density lipoprotein	(Nanashima et al., 2021)
Renal system	Juice	Higher citric acid and oxalic acid production and reduced neutrophils (PMN leukocytes)	
Improvement in fat oxidation	Extract	During fast jogging enhanced fat oxidation in whole body	(Hiles et al., 2020
		Increased lipid metabolism	(Song et al., 2021)
Wounds	Polyunsaturated fatty acid	Enhanced anti‐inflammatory compounds and reduced prothrombotic compounds in TPN administration	(Calder, 2009)
Gut microbiota improvement	Supplementation	Treatment shifts the gut microbiome of a female mice in an age‐dependent adjustment	(Cao et al., 2020)
		Increased bacterial phylotype in the gut	(Song et al., 2021)
Pulmonary system	Leaf extract	Reduced inflammation and restricted migration of neutrophilic cells	(Garbacki et al., 2004)
Lowering in postprandial blood sugar	Powder	Demonstrated suppression of α‐amylase and α‐glucosidase and anthocyanins seem to control postprandial hyperglycemia	(Barik et al., 2020; Hui et al., 2020)
	Extract	Lower blood glucose levels, enhanced glucose metabolism, regulate insulin levels and free‐living postprandial glucose excesses in individuals with obesity or overweight	(Nolan et al., 2021)
Tumors	Peel extract	Decreased tumor weight, reduced the size, number, and volume of hepatocyte nodules	(Bishayee et al., 2011; Takata et al., 2007)
		Decreased the area and number of GGT‐positive foci, decrease the expansion of HSP70, HSP90, COX‐2 and NF‐KB, degraded protein and lipid oxidation	
Enhancement in mental function (Cognition)	Concentrate	Enhance mental function in athletic population	(Gibson et al., 2020)
	Juice	Impact on the monoamine axis in humans to enhance cognitive function	(Watson et al., 2020)

### 6.1 Antimicrobial activity

Various scholars recently examined the antibacterial effects of dietary plant bioactive components on pathogenic microbes in extreme detail (Abouzeed et al., 2018). Analyzing the polysaccharides available in blackcurrant seeds, Lengsfeld et al. (2004) observed that Helicobacter pylori stuck to the gastrointestinal mucosa less frequently. Crude polysaccharides from blackcurrant seeds possess anti‐adhesive activities as they contain acidic increased‐molecular weight Galatians that can connect to *H*. *pylori* receptors and prevent bacteria from sticking to the digestive mucosa. In contradiction, it indicated that blackcurrant concentrate had an excellent antibacterial potential in inhibiting the colonization of oral *Porphyromonas gingivalis*, *Aggregatibacter actinomycetemcomitans*, *Actinomyces naeslundii*, and *Fusobacterium nucleatum* (Kranz et al., 2020). An organic substance from blackcurrant is believed to possess potential as a technique for preventing periodontitis. From the outcomes of a six‐week in vivo investigation (using male Sprague Dawley mice), Paturi et al. (2018) suggested that blackcurrant anthocyanins may change the gut bacterial microflora. Blackcurrant extract and anthocyanins have been reported to suppress peridonopathogenic proteinases like *Treponema denticola*, *Tannerella forsythia*, and *Porphyromonas gingivalis* used to collect valuable nutrients from their surroundings (Santos et al., 2011). Blackcurrant fruit extract's anti‐herpetic properties were evaluated in vitro. Through the suppression of protein synthesis during the preliminary phases of infection, blackcurrants have been demonstrated to reduce the bonding of *herpes simplex* virus type 1 to the cell barriers, in addition to the plaque accumulation of herpes simplex virus type 1 and 2 and the *Varicella‐Zoster* virus. Blackcurrant anthocyanins have been discovered to possess powerful antiviral effects against influenza viruses A and B (Suzutani et al., 2003).

### 6.2 Anti‐diabetic activity

The maintenance of postprandial hyperglycemic, one of these recently found therapeutic benefits, is especially interesting and critical because of its association to type 2 diabetes mellitus (T2DM). Hui et al. (2020) evaluated the anti‐amylase and anti‐glucosidase characteristics of blackcurrant extract using pastes comprising blackcurrant powder. Their research demonstrated that a blackcurrant combination with bran of oat might be a source of bioactive compounds with anti‐hyperglycemic ability. The intensity of starch decomposition and the level of reducing sugar discharge from the mixture during in vitro analysis of both were drastically decreased, based on researcher's further analysis of starch ingestion of this paste including blackcurrant extract (Hui et al., 2021). This research showed that blackcurrant powder fortification might result in the production of starchy food with decreased glycemic content. The observation that anthocyanins of blackcurrant decreased postprandial hyperglycemia by suppressing glucosidase action was mostly in accordance with this research (Barik et al., 2020). According to Barik et al. (2020), the predominant phenolics that altered salivary amylase action included blackcurrant anthocyanins and other phenolics. Collectively, these experiments concluded that adding blackcurrant powder to cereal products could significantly decrease the probability of getting T2DM. Moreover, the absence of additional in vivo verification puts these conclusions in a specific context.

### 6.3 Antioxidant activity

Various studies present data supporting blackcurrant's capability to function as antioxidant (Salobir et al., 2010). Evidence from many other investigations found that hydrogen peroxide (H_2_O_2_) and NO are neutralized along with the inhibition of the development of lipid and protein peroxidation (Viljanen et al., 2004). Furthermore, it has been discovered that the constituents of blackcurrant considerably stimulate the antioxidant enzymes like glutathione (GSH) peroxidase and superoxide dismutase through an undetermined process. The phenolic and anthocyanin concentrations of blackcurrant berry as a whole are significantly responsible for their particular antioxidant activities (Ehala et al., 2005). According to the cultivar, the harvest season, the stage of maturation, and the specific plant part utilized, this composition might differ greatly. Significant concentrations of vitamin C provide blackcurrants extra intrinsic antioxidant properties they generally possess, even though the phenolic level is believed to contribute to a more powerful to antioxidant capacity than organic vitamins (Borges et al., 2010). The pH of the environment has an impact on the antioxidant capacity of the anthocyanins in the blackcurrants during oral intake, may be as a result of change in its protropic balance. Highest concentrations of free radical scavenging action were identified at pH values between 6.0 and 7.0, which are a bit more acidic as compared to human serum (Estévez et al., 2010). This indicates that the antioxidant action of anthocyanins can differ based on where they are located in the body. During 2 h of intake, research on blackcurrant juice in humans demonstrated a rise in serum sulfahydryl group levels. A 94% suppression of copper ion‐induced decrease density lipoproteins (LDL) oxidation was reported in additional in vitro investigations (Rosenblat et al., 2010).

### 6.4 Anti‐inflammatory and anti‐obesity activity

The anti‐inflammatory properties of blackcurrant polyphenols have gained a lot of attention recently. Studies with obesity‐related inflammation have shown interesting outcomes for the management of blackcurrant (Kim et al., 2016). In animal studies, diet‐induced obesity is a well‐studied source of minor systematic inflammation. The impacts of blackcurrant extract were evaluated by Lee et al. (2019) in treated mice with weight‐induced non‐alcoholic steatohepatitis. Based on the studies, feeding mice a high‐fat/high‐sucrose diet for 24 weeks also including 6% whole blackcurrant powder reduced pro‐inflammatory M1 macrophage accumulation in the liver. Furthermore, blackcurrant extract considerably decreases liver weights and formation of triacylglycerol in the liver. Additionally, flow cytometry evaluation of mice treated with blackcurrant extract and subjected to inflammation showed a substantial decrease in overall population of invasive immune cells in the mice's lungs (Shaw et al., 2017). Other in vitro research observations have indicated that blackcurrant polyphenolic components have therapeutic effects in lowering lung inflammation and possibility of developing allergic asthma (Hurst et al., 2010). In an intensive in vitro analysis of inflammation, Ferrari et al. (2016) indicated that cyaniding‐3‐O‐glucoside has preventive effects. Olejnik et al. (2016) also observed consistent outcomes. In Caco‐2 cells, hydrolysis of a freeze‐dried blackcurrant extract (1 mg/mL) decreases the levels of IL‐8 (by 54%) and COX2 (by 17%).

The nuclear factor‐kappa‐light‐chain‐improved of activated B cells (NF‐B) system is mainly responsible for the well‐known anti‐inflammation of blackcurrant anthocyanins. As a transcription factor, NF‐B modulates a variety of genes implicated in immunological process and inflammation. These included chemokines, cytokines that cause inflammation, adhesion molecules, and inactivating enzymes, like cyclooxygenases and inducible nitric oxide synthase (iNOS) (Moynagh, 2005). In several macrophage cell lines, NF‐B activity was decreased by blackcurrant anthocyanins in reaction to LPS (Lee et al., 2014; Lyall et al., 2009). A recent research demonstrated that bilberry and blackcurrant intake lowered the production genes (pro‐inflammatory) associated with NF‐B system in whole diet samples taken from individual participants with gastric syndrome (Aboonabi & Aboonabi, 2020).

### 6.5 Anti‐cancer activity

Research on blackcurrant's possible influence on cancer cells was not initiated until now. It has been proved that blackcurrant extract inhibits the progression of colon cancer, breast cancer cells HT‐29/ HCT‐166, and human promyelocytic leukemia cells MCF‐7 BCE (Wu et al., 2007). Although becoming the second major prevalent cause of cancer‐associated deaths globally, it is questionable that blackcurrant extract can efficiently suppress the gastrointestinal cancer cells (Houghton et al., 2002). The production of blackcurrant extract and its impact toward free radicals in addition to stomach cancer SGC‐7901 cells were analyzed in the latest research. Various researchers have utilized a spectrum of tumor cells with human history to evaluate the anti‐cancer effects of blackcurrants. Complete fruit extracts have been found to prevent the expansion of MCF‐7 breast cancer cells and HT29 colon cancer cells (Olsson et al., 2004). The inhibition of p21WAF1 pathway was recognized as the underlying principle in a systematic review that represented the anti‐proliferative impact of blackcurrant powder toward HT29 colon cancer cells (Wu et al., 2007). McDougall et al. (2008) identified a related growth reduction potential of an extract produced by employing the entire fruit toward HeLa cervical cancer cells. The foremost anti‐proliferative action over various types of cancer was achieved by blackcurrant juice. Blackcurrant juice's chemotherapeutic impact on prostate cancer cells is attributed to its mechanistic action, involving cell cycle disruption, decreased TNF‐induced stimulation of cyclooxygenases (COX‐2) activity, and diminished TNF‐induced NF‐κB‐dependent reporter gene transcription (Boivin et al., 2007). Cassis polysaccharide (CAPS), a polysaccharide‐rich compound obtained from blackcurrant juice, has exhibited cytotoxicity over Ehrlich ascites tumor cells (Takata et al., 2005). Different human cancer cell models, especially Caco‐2, HT‐29, and HCT 116, were analyzed extensively for their potential to suppress the proliferation of blackcurrant pressed leftover extract prepared after extraction at different temperatures. Against all tested cell strains, all the isolates exhibited dose‐dependent reduction of cell function. When the extract prepared at 90 C was administered to HT‐29 cells, a significant enhancement of apoptosis was shown (Holtung et al., 2011).

## 7 CONCLUSION

Recent investigations have confirmed that blackcurrant contains a number of bioactive components that are extracted using different extraction techniques. The extracts from the blackcurrant fruit, especially from the peel, seed, leaves, and pulp, have been found to contain phytochemicals that have medicinal and nutritional properties in several in vitro and in vivo studies. In order to improve the industrial processing of blackcurrant as a functional or therapeutic food product, it is necessary to conduct further examination into its nutritional and physiological potential.

## AUTHOR CONTRIBUTIONS

**Afaf Ejaz:** Validation (equal); writing – original draft (equal); writing – review and editing (equal). **Sadaf Waliat:** Formal analysis (equal); validation (equal); writing – review and editing (equal). **Muhammad Afzaal:** Supervision (equal); validation (equal); writing – review and editing (equal). **Farhan Saeed:** Formal analysis (equal); supervision (equal); validation (equal). **Aftab Ahmad:** Data curation (equal); validation (equal). **Ahmad Din:** Formal analysis (equal); validation (equal). **Huda Ateeq:** Investigation (equal); validation (equal). **Asma Asghar:** Data curation (equal); validation (equal). **Yasir Abbas Shah:** Data curation (equal); formal analysis (equal); writing – review and editing (equal). **Ahmad Rafi:** Data curation (equal); validation (equal). **Mahbubur Rahman Khan:** Data curation (equal); validation (equal).

## FUNDING INFORMATION

The authors declare that no funds, grants, or other support were received during the preparation of this manuscript.

## CONFLICT OF INTEREST STATEMENT

The authors have no relevant financial or non‐financial interests to disclose.

## ETHICS STATEMENT

This article does not contain any studies with human participants or animals performed by any of the authors.

## INFORMED CONSENT

For this type of study, formal consent is not required.

## CONSENT TO PARTICIPATE

Corresponding and all the co‐authors are willing to participate in this manuscript.

## Data Availability

Even though adequate data have been given in the form of tables and figures, all authors declare that if more data are required, then the data will be provided on a request basis.
